# Comment on Santana-Bejarano et al. NRP1 and GFAP Expression in the Medulloblastoma Microenvironment: Implications for Angiogenesis and Tumor Progression. *Cancers* 2025, *17,* 2417

**DOI:** 10.3390/cancers18060913

**Published:** 2026-03-12

**Authors:** Rafael Roesler, Gustavo R. Isolan

**Affiliations:** 1 Department of Pharmacology, Institute for Basic Health Sciences, Federal University of Rio Grande do Sul, Porto Alegre 90035-003, Brazil; 2 Cancer and Neurobiology Laboratory, Experimental Research Center, Clinical Hospital (CPE-HCPA), Federal University of Rio Grande do Sul, Porto Alegre 90035-003, Brazil; 3 National Science and Technology Institute for Children’s Cancer Biology and Pediatric Oncology—INCT BioOncoPed, Porto Alegre 90035-003, Brazil; 4 Center for Biotechnology, Federal University of Rio Grande do Sul, Porto Alegre 91501-970, Brazil; 5 Graduate Program in Principles of Surgery, Mackenzie Evangelical University, Curitiba 80730-000, Brazil; 6 The Center for Advanced Neurology and Neurosurgery (CEANNE), Porto Alegre 90560-010, Brazil

We read with great interest the recent study by Santana-Bejarano et al., entitled “*NRP1 and GFAP Expression in the Medulloblastoma Microenvironment: Implications for Angio-genesis and Tumor Progression*” [1]. Their work highlights several important aspects of research on novel molecular targets in medulloblastoma (MB), including the relevance of examining target expression not only in tumor cells but also in non-tumoral components of the microenvironment, such as tumor-associated microglia/macrophages (TAMs), endothelial cells, and astrocytes. Associations of the density of cells positive for neuropilin-1 (NRP1) with patient survival in the authors’ own cohort failed to reach statistical significance, likely because of the small sample size (39 patients), although there was a clear trend towards an association between high NRP1+ cell content and poorer survival in the sonic hedgehog (SHH) and non-Wingless (WNT)/SHH MB subgroups.

As noted by Santana-Bejarano et al., these observations contrast with those from our study focusing on *NRP1* gene transcription levels in a large MB tumor cohort. Consistently with results presented by Santana-Bejarano et al., we found significantly higher *NRP1* expression in SHH MB compared to other subgroups, particularly non-WNT/SHH (Group 3 and Group 4). This finding was independently confirmed in a second dataset. However, our data showed that low *NRP1* expression was associated with shorter overall survival (OS) when tumors from all subgroups were evaluated collectively, and specifically in SHH and Group 3 tumors [2]. It is important to point out that associations between gene expression and patient survival do not by themselves implicate causality. Such associations may suggest potential utility as prognosis biomarkers, but our study did not address whether NRP1 plays a direct functional role in MB progression.

We believe that addressing possible explanations for the apparent discrepancy between our findings and those of Santana-Bejarano et al. is essential. A key difference lies in the methodological approaches: while our study assessed gene expression by mRNA levels, Santana-Bejarano et al. used immunohistochemistry (IHC) to detect the NRP1 protein and determine the density of cells expressing NRP1. It is well established that mRNA expression does not necessarily correlate with protein content, due to multiple layers of post-transcriptional and post-translational regulation that influence protein levels independently of gene expression [3,4,5]. Advancing the validation of NRP1 as a prognostic biomarker in MB will require integrating both mRNA and protein-level analyses, ideally distinguishing expression in tumor cells from that in diverse cellular components of the tumor microenvironment.

## Abbreviations

The following abbreviations are used in this manuscript:

IHC	Immunohistochemistry
MB	Medulloblastoma
NRP1	Neuropilin-1
OS	Overall survival
SHH	Sonic hedgehog
TAM	Tumor-associated microglia/macrophage
WNT	Wingless
